# Limited Contribution of Hepatic Progenitor Cells to Hepatocyte Replenishment After Acetaminophen Overdose

**DOI:** 10.3390/biology15211908

**Published:** 2026-10-22

**Authors:** Diego Paine-Cabrera, Giselle Sanchez-Guerrero, Dakota Robarts, Manasi Kotulkar, Udayan Apte

**Affiliations:** Department of Pharmacology, Toxicology, and Therapeutics, University of Kansas Medical Center, 3901 Rainbow Blvd., MS1018, Kansas City, KS 66160, USA; dpainecabrera@kumc.edu (D.P.-C.); gesanche@ncsu.edu (G.S.-G.);

**Keywords:** hepatic progenitor cells, liver regeneration, acetaminophen

## Abstract

Liver regeneration is critical determinant of survival following acetaminophen (APAP) overdose, driven primarily by proliferation of surviving hepatocytes. Hepatic progenitor cells (HPCs) are bipotential cells activated when hepatocyte proliferation is impaired. The contribution of HPCs to liver regeneration after APAP overdose remains unclear. This study investigated the contribution of HPCs to hepatocyte repopulation when regeneration is impaired after severe APAP overdose using a lineage-tracing mouse model. Two-month-old male C57BL/6J mice were fasted overnight and then administered a 600 mg/kg overdose of APAP (600APAP). For lineage tracing, male ROSA^mT-*mG*^ mice were injected with AAV8-TBG-Cre to label hepatocytes. Two weeks after AAV administration, mice were fasted overnight and then administered a 600 mg/kg overdose of APAP. Liver and blood samples were collected multiple times. C57BL/6J mice treated with severe acetaminophen overdose (600APAP) showed substantial liver injury, with peak serum ALT and hepatic necrosis at 72 h, followed by progressive recovery. HPCs activation was evidenced by increased expression of EpCAM, A6. CK19, cholangiocyte marker, increase during the recovery phase. APAP administration to ROSA^mT-*mG*^ mice induced liver injury and subsequent regeneration and recovery comparable to wild type mice. Detection of HNF4α/tdTomato double-positive cells, hepatocytes originating from a non-parenchymal source, were detected after injury but represented only 0.3–0.6% of total hepatocytes. Taken together, this study indicates that despite an increase of HPC/cholangiocyte markers, HPC/cholangiocyte transdifferentiation contributes minimally to hepatocyte replenishment during an impaired regeneration after severe APAP-induced liver injury.

## Simple Summary

Acetaminophen overdose is a leading cause of acute liver failure, and recovery depends on the liver’s ability to replace damaged cells. However, when liver injury is severe, this process can be impaired. This study investigated a population called hepatic progenitor and biliary cells with the potential to generate different liver cell types, contributing to hepatocyte replacement after severe acetaminophen overdose. Using a linage-tracing mouse model, where the origin of new hepatocyte could be tracked, we found substantial liver injury followed by progressive recovery. Although markers associated with liver progenitor and bile duct cells increased during injury and recovery, only 0.3–0.6% of hepatocytes originated from other cell populations. These findings indicate that liver progenitor cells make only a minimal direct contribution to replacing hepatocytes after severe acetaminophen-induced liver injury. Understanding which cells are responsible for liver repair may help guide future strategies to improve recovery and survival in patients with severe acetaminophen overdose.

## 1. Introduction

Liver regeneration is a remarkable process that permits the liver to replenish dead tissue after surgical resection, chemical injury, and dietary stress [1,2,3]. At the center of liver regeneration is proliferation of parenchymal (hepatocytes) as well as non-parenchymal cells, which restore the liver mass, architecture, and function [1,2,3]. Hepatic progenitor cells (HPCs), also referred to as liver progenitor cells (LPCs) or oval cells in rodents, are bipotential epithelial cells capable of differentiating into both hepatocytes and cholangiocytes [4,5]. These cells reside within the canals of Hering and periportal regions, where they remain quiescent during homeostasis but can be activated in response to severe liver injury [4,5,6]. The activation of HPCs is a characteristic of chronic liver diseases. HPCs contribute to liver regeneration when the proliferative capacity of mature hepatocytes is impaired or insufficient to restore liver function [4,5,7].

Acetaminophen (APAP) overdose is the leading cause of acute liver failure (ALF) in the United States [8,9,10]. Extensive studies have shown that liver regeneration plays a critical role in survival after APAP overdose [11,12,13,14]. APAP-induced acute liver injury results in necrotic cell death, which is followed by liver regeneration and recovery in a dose-dependent manner [15]. Following moderate overdose, the liver undergoes regeneration characterized by timely and effective hepatocyte proliferation, where dead cells are replaced predominantly by the proliferation of surviving hepatocytes [11,15]. However, following severe overdose, liver regeneration is delayed and inhibited, resulting in progression of liver injury and acute liver failure (ALF) [15]. Studies have shown that the regenerative response after APAP overdose is dose-dependent in mouse models [15,16]. Following administration of 300 mg/kg APAP, liver regeneration and recovery occur, while 600 mg/kg APAP treatment results in impaired regeneration, ALF, and mortality [11,15,16].

Previous studies have shown that following chronic or severe injury, liver regeneration is inhibited, leading to HPCs activation, proliferation, and transdifferentiation into hepatocytes [5,17,18]. However, the contribution of HPCs to liver regeneration after severe APAP overdose has not been evaluated in detail. In this study, we analyzed the contribution of HPCs to hepatocyte replacement after a severe APAP overdose (600 mg/kg). Our results describe an increase in the HPC/cholangiocyte markers; however, the transdifferentiation into hepatocyte during liver recovery is limited.

## 2. Materials and Methods

### 2.1. Animals, Treatments, and Tissue Collection

All animal procedures were conducted according to the protocols approved by the Institutional Animal Care and Use Committee (IACUC) of the University of Kansas Medical Center (KUMC) (protocol code IPROTO-23-09-346; approval date: 13 March 2025), in compliance with the ARRIVE guidelines. Mice were maintained in AAALAC-accredited facilities under a standard 12 h light/dark cycle with ad libitum access to chow and water. Eight-week-old male C57BL/6J mice (Strain #000664, The Jackson Laboratory, Bar Harbor, ME, USA) were fasted overnight and injected intraperitoneally with 600 mg/kg of body weight of APAP (Sigma, St. Louis, MO, USA) dissolved in warm 0.9% saline. For the linage-tracing experiment, Eight-week-old male B6.129(Cg)-*Gt(ROSA)26Sor^tm4(ACTB-tdTomato,-EGFP)Luo^*/J (ROSA^mT-*mG*^ mice; Strain #007676, The Jackson Laboratory, Bar Harbor, ME, USA) received a single intraperitoneal injection of AAV8-TBG-Cre (2 × 10^11^ genome copies [GC]/mouse, VB1724, Vector Biolabs, Newark, CA, USA). Two weeks later, mice were fasted overnight and injected intraperitoneally with 600 mg/kg of APAP. At the beginning of the survival study, 10 to 15 mice of each strain were allocated to each APAP time point, whereas 4 mice per strain were allocated to the untreated (0 h) group. Due to APAP-induced mortality, the actual number of animals analyzed at each time point ranged from 4 to 5; detailed animal numbers are provided in Table 1. At the indicated point, survival mice were anesthetized with isoflurane and euthanized by exsanguination followed by cervical dislocation. Blood samples were incubated at room temperature for 30 min, then centrifuged at 5000 RCF for 10 min at 4 °C to obtain serum and stored at −80 °C until biochemical analysis. Livers were excised immediately following euthanasia. Liver tissues were fixed in 10% neutral-buffered formalin for histological analysis, while the remaining tissues were snap-frozen in liquid nitrogen and stored at −80 °C for subsequent analyses.

### 2.2. Biochemical Measurements

Serum alanine aminotransferase (ALT) activity was quantified using the ALT (SGPT) Liquid Reagents (A7526-625, HORIBA/Pointe Scientific, Canton, MI, USA) using a kinetic UV spectrophotometric assay based on the modified IFCC method. ALT activity was determined by measuring the rate of NADH oxidation as a decrease in absorbance at 340 nm.

### 2.3. Histology and Immunohistochemistry

Formalin-fixed paraffin-embedded liver sections (5 µm thick) were used for hematoxylin and eosin (H&E) staining [19] using a Leica CV 5030 autostainer (Leica Microsystems, Wetzlar, Germany). Immunohistochemistry was performed on 5 µm paraffin-embedded liver sections. Antigen retrieval was conducted in boiling citrate buffer (pH 6.0) for 30 min. Sections were blocked with 5% normal goat serum (005-000-121, Jackson ImmunoResearch Inc., West Grove, PA, USA) for 1 h at room temperature and incubated overnight at 4 °C with primary antibodies (Table 2). Following incubation with biotinylated secondary antibodies (Table 2) for 30 min at room temperature, target proteins were detected using the VECTASTAIN Elite ABC-HRP Kit (PK-4000), together with the ImmPACT DAB (SK-4105) or ImmPACT VIP (SK-4605) substrates (all from Vector Laboratories, Newark, CA, USA). Slides were counterstained with hematoxylin (GHS316, Sigma-Aldrich, Lenexa, KS, USA). Images were acquired using an Olympus DP74 color camera mounted on an Olympus BX51 microscope (Olympus Corporation, Tokyo, Japan) and analyzed with CellSens software (version 2.3; Olympus Corporation, Tokyo, Japan). CK19 quantification was performed on 200× magnification images, and positive cells were manually quantified using the Counting Panel tool from QuPath (version 0.7.0; open-source software; https://qupath.github.io/, Edinburgh, UK).

### 2.4. Immunofluorescence

Frozen OCT-embedded liver sections (5 µm thick) were fixed using 10% formalin for 10 min, permeabilization was performed using 0.1% Triton X-100 over 15 min, and samples were washed three times with 1× PBS for 5 min each. The liver sections were blocked in 10% normal serum (donkey or goat 017-000-121, 005-000-121, Jackson Immuno Research Inc., West Grove, PA, USA) in PBS for 1 h at room temperature. The primary antibodies described in Table 3 were incubated at 4 °C overnight. PBS was used to wash 3 times and the fluorophore-conjugated secondary antibodies described in Table 3 were added and incubated for 1 h at room temperature. Finally, Mounting Medium without DAPI (H-1700-2, Vector Laboratories, Newark, CA, USA) or Mounting Medium with DAPI (H-1200-10, Vector Laboratories, Newark, CA, USA) were added to cover the slide. Photomicrographs were captured using an Olympus DP74 color camera mounted on an Olympus BX51 microscope (Olympus Corporation, Tokyo, Japan) with CellSens software (version 2.3; Olympus Corporation, Tokyo, Japan). Quantification of A6- and EpCAM-positive cells was performed on 200× images using QuPath (version 0.7.0; open-source software; https://qupath.github.io/, Edinburgh, UK). Cell segmentation was based on nuclear detection in the DAPI channel using the Cell Detection tool, with cell boundaries estimated by simulated cell expansion (10–20 µm). Independent single-measurement object classifiers were generated for each marker. Cells were classified according to the mean intensity of the green channel (Cy2) for A6 and EpCAM, with positivity thresholds empirically determined by visual inspection. For tdTomato reporter experiments, tdTomato- and HNF4α-positive cells were manually quantified using the Counting Panel tool from QuPath (version 0.7.0).

### 2.5. Statistical Analysis

Data are shown as mean ± standard deviation (SD) in bar graphs and result descriptions. Statistical analysis and graph generation were made using GraphPad Prism 10 (version 10; GraphPad Software, Boston, MA, USA). Between 3–5 survival mice per group were analyzed. Comparisons between groups were performed using nonparametric statistical analyses. Differences between individual time points were assessed using the Mann–Whitney U test. A *p*-value < 0.05 was considered statistically significant.

## 3. Results

### 3.1. APAP Overdose Induces Severe Liver Injury

**Figure 1:**
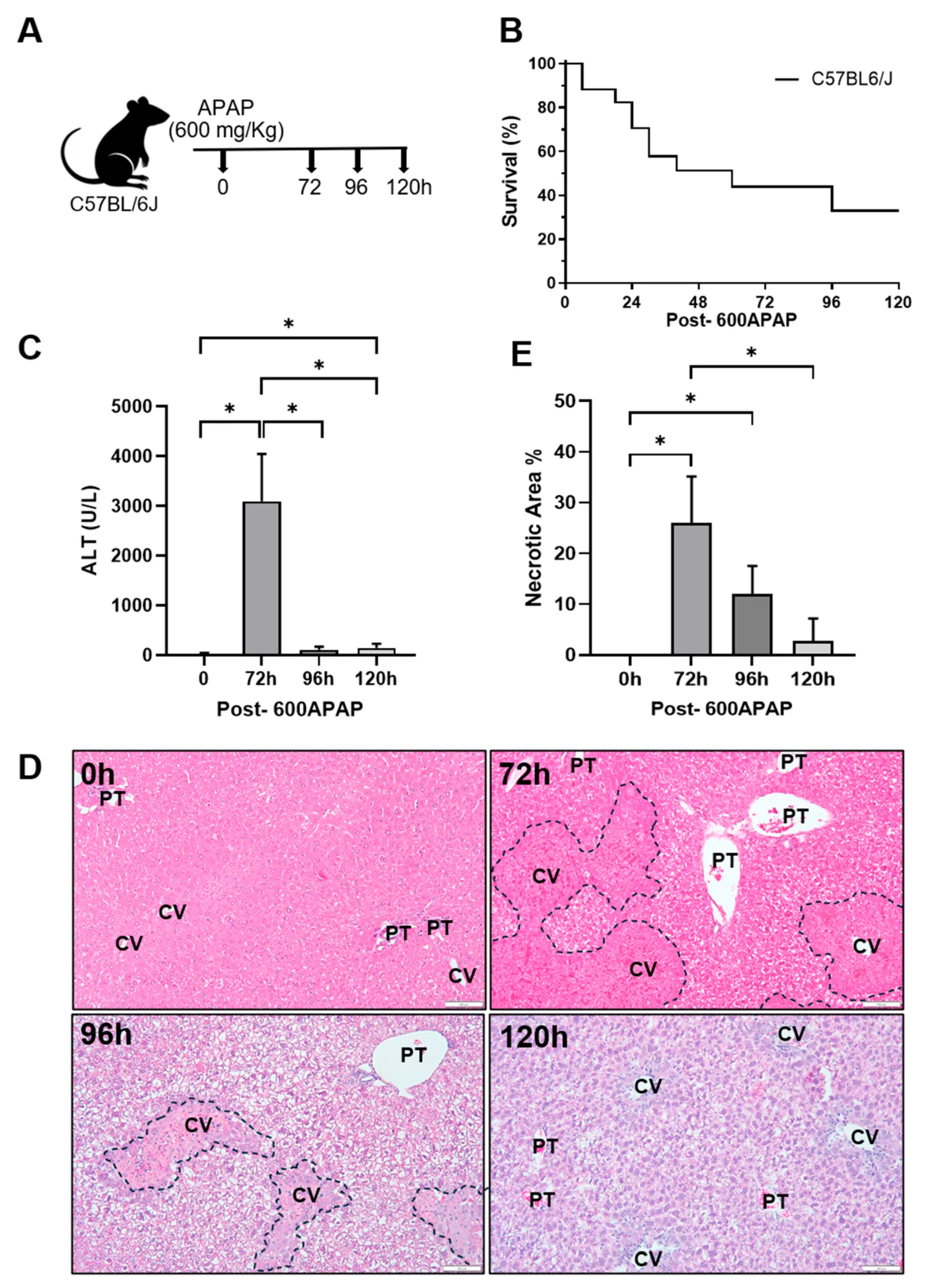
Severe liver injury after 600APAP in wild type mice. (**A**) Experimental design. (**B**) Kaplan–Meier survival analysis. (**C**) Serum ALT activity. (**D**) Representative micrographs of H&E-stained liver sections. Black dashed lines indicate necrotic areas. Portal trials (PT) and central veins (CV) are indicated. Scale bar = 100 µm. (**E**) Quantification of liver necrosis from H&E-stained liver sections. Samples analyzed per group: *n* = 4 at 0 h, *n* = 4 at 72 h, *n* = 5 at 96 h, and *n* = 4 at 120 h. Bars represent mean ± SD, and * *p* < 0.05.

We treated the C57BL6/J (wild type) mice with an overdose of 600 mg/kg of APAP (600APAP). Liver tissue and serum were collected to assess injury in a timeline manner (Figure 1A). At 120 h, we observed 33% survival after 600APAP (Figure 1B). Surviving mice exhibited significant liver injury, as demonstrated by elevated serum ALT levels at 72 h after the APAP overdose, which rapidly declined at the baseline at 96 h and 120 h (Figure 1C). Consistent with these findings, histological analysis by H&E staining revealed 25.92 ± 7.9% hepatic necrosis at 72 h (Figure 1D,E). Necrotic areas decreased to 9.8 ± 5.4% at 96 h and at 120 h after 600APAP the liver tissue was almost recovered (2.79 ± 3.88% of necrotic areas) (Figure 1D,E). These results show that 600APAP causes hepatic injury, followed by liver recovery at later time points.

### 3.2. Hepatic Progenitor Cells Markers Are Increased After Severe APAP Overdose

**Figure 2:**
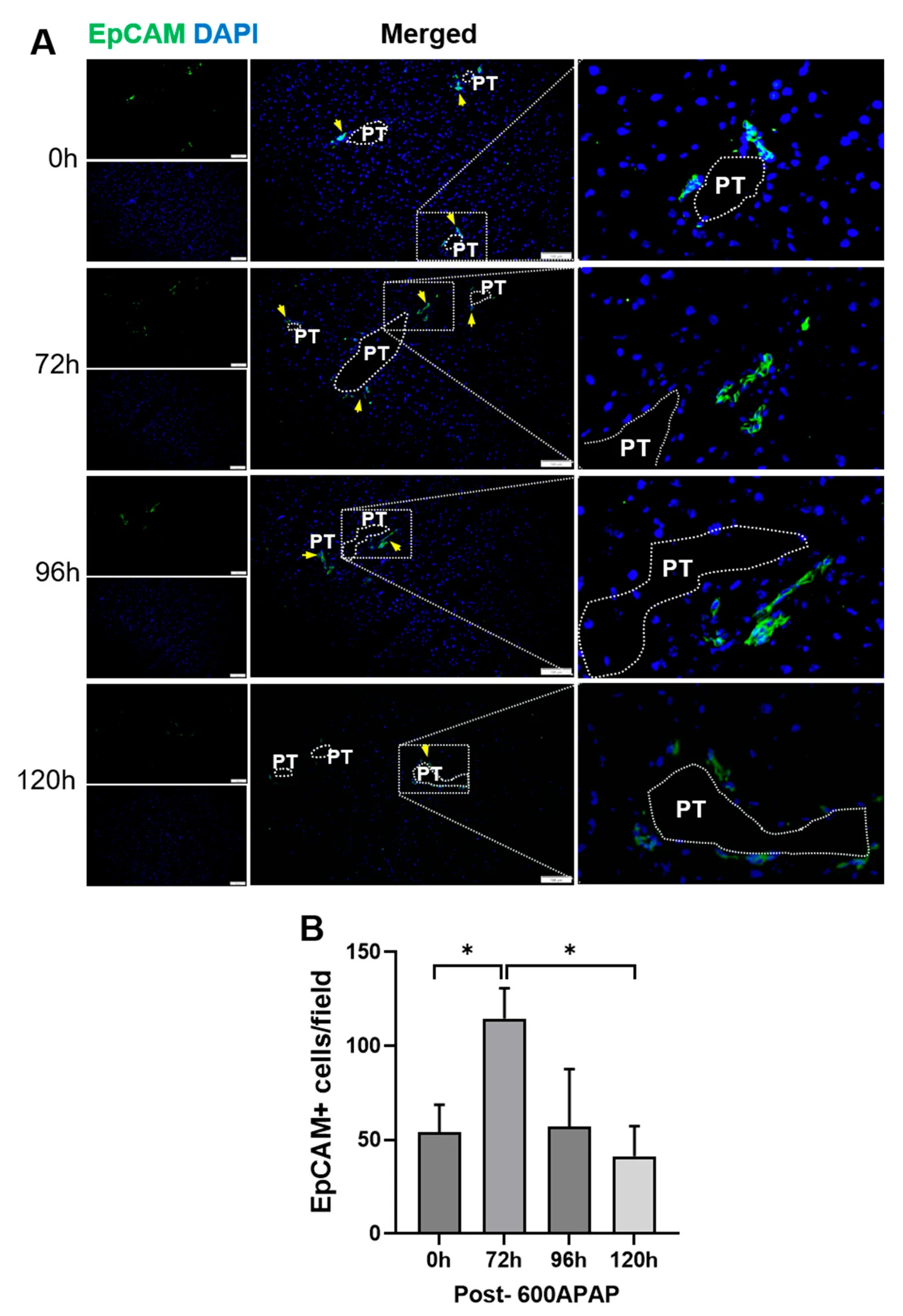
EpCAM expression increases after 600APAP in wild type mice. (**A**) Representative photomicrographs of EpCAM immunofluorescence staining (green) in liver sections. Nuclei are counterstained with DAPI (blue). Scale bar = 100 µm. Dotted white line represents portal vein area, PT: portal trial, and yellow arrows indicate positive cells, including dotted white square portal trials region magnification (20,000× magnification) (**B**) Quantification of EpCAM-positive cells per field in liver sections. Samples analyzed per group: *n* = 4 at 0 h, *n* = 4 at 72 h, *n* = 3 at 96 h, and *n* = 4 at 120 h. Bars represent mean ± SD, and * *p* < 0.05.

**Figure 3:**
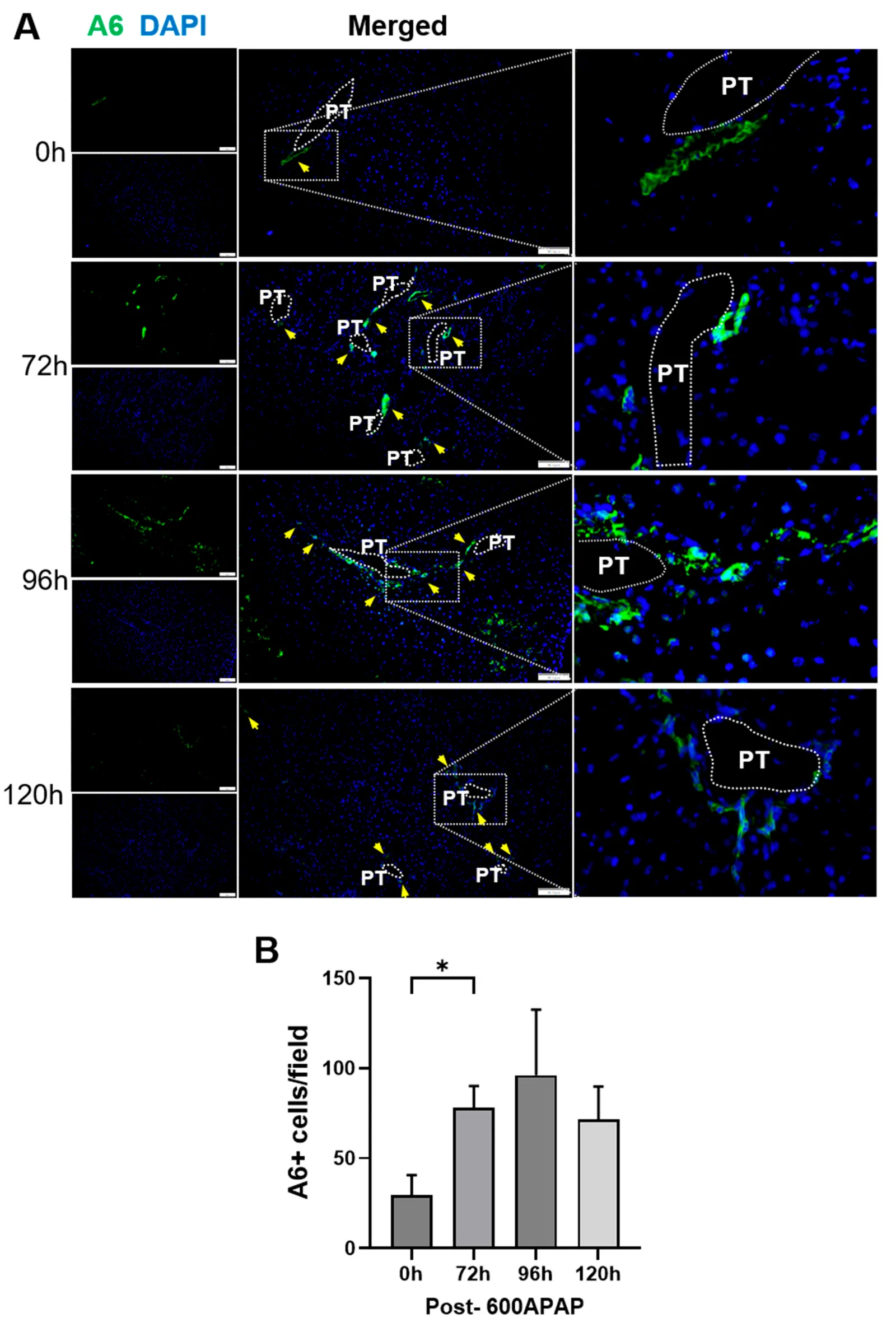
A6 expression increases after 600APAP in wild type mice. (**A**) Representative micrographs of A6 immunofluorescence staining (green) in liver sections. Nuclei are counterstained with DAPI (blue). Scale bar = 100 µm. Dotted white line represents portal vein area, PT: portal trial, and yellow arrows indicate positive cells, including dotted white square portal trials region magnification (20,000× magnification) (**B**) Quantification of A6-positive cells per field in liver sections. Samples analyzed per group: *n* = 4 at 0 h, *n* = 4 at 72 h, *n* = 3 at 96 h, and *n* = 4 at 120 h. Bars represent mean ± SD, and * *p* < 0.05.

When hepatocytes are unable to restore liver function, hepatic progenitor cells (HPCs) are activated to support liver regeneration. To confirm HPC activation after APAP overdose, we assessed the expression of the HPC markers EpCAM and A6 by immunofluorescence. Cytokeratin 19 (CK19), a biliary cell and HPC marker, was detected using immunohistochemistry over time. EpCAM-positive cells increased to 114.58 ± 13.98 cells per field in the periportal region by 72 h and returned to baseline levels (54 ± 12.7 cells per field) by 96 h and 120 h after 600APAP (Figure 2). Similarly, A6-positive cells increased from 26.66 ± 9.49 positive cells at baseline to 77.87 ± 12.12 cells per field at 72 h after 600APAP and remained elevated through 120 h (Figure 3).

**Figure 4:**
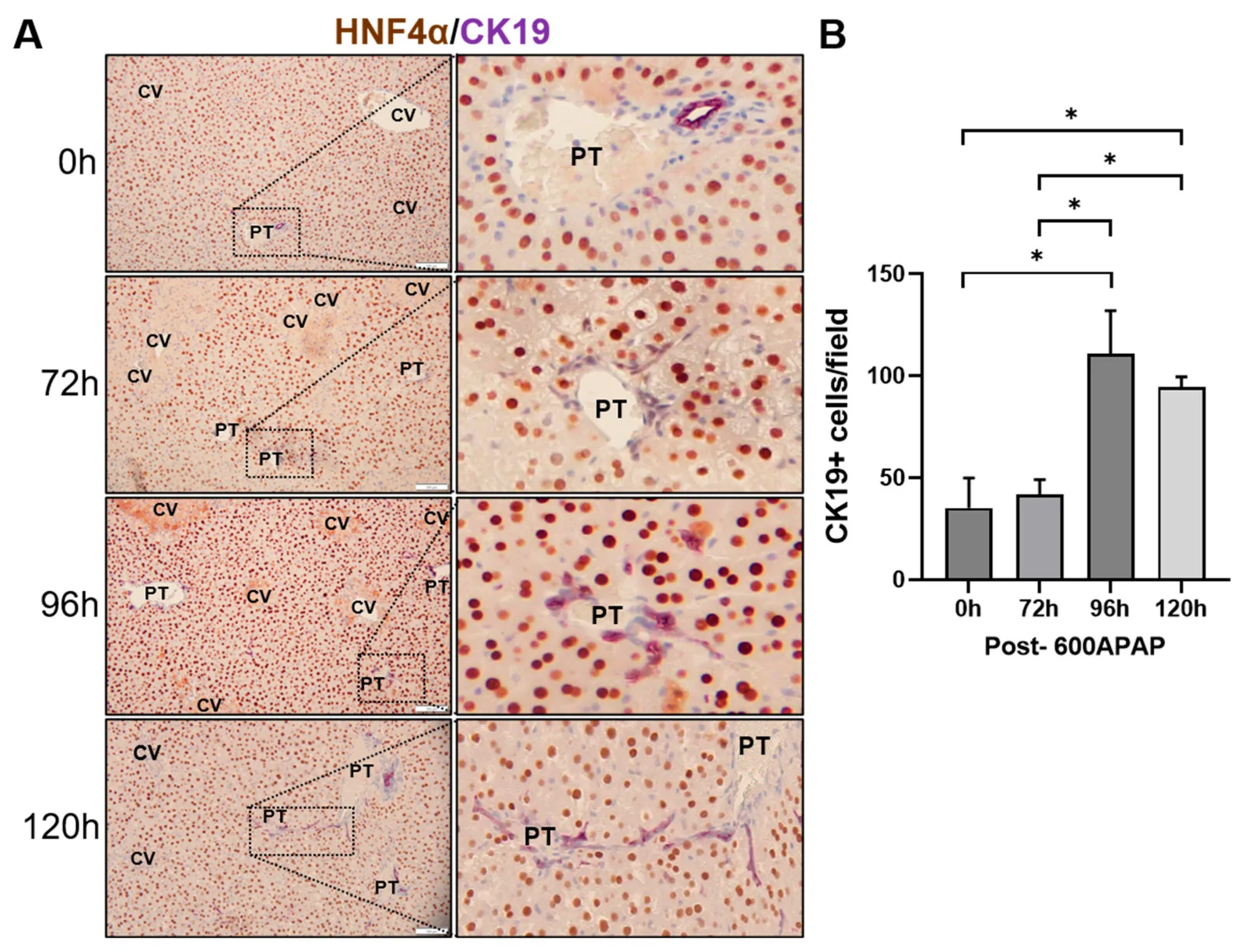
CK19 expression increases after 600APAP in wild type mice. (**A**) Representative micrographs of CK19 (purple) and HNF4α (brown) co-immunohistochemical staining in liver sections. Portal trials (PT) and central veins (CV) are indicated. Scale bar = 100 µm. Including portal trials region magnification (15,000× magnification). (**B**) Quantification of CK19-positive cells per field in liver sections. Samples analyzed per group: *n* = 4 at 0 h, *n* = 4 at 72 h, *n* = 4 at 96 h, and *n* = 4 at 120 h. Bars represent mean ± SD, and * *p* < 0.05.

We performed the double immunohistochemical analysis of CK19, marker for biliary epithelial cells, and HNF4α markers for hepatocyte. Dual staining shows nuclear HNF4α in brown and cytoplasmic CK19 in purple (Figure 4A). Non-treated animals (0 h) exhibit CK19 expression restricted to the biliary ducts. However, after acute injury by 600APAP, its expression increases and extends into the sinusoids, presenting as elongated cells. CK19 expression is significantly increased, from 40.33 ± 10.66 positive cells at baseline to 118.89 ± 14.1 positive cells by 96 h post-APAP, and this elevated level is maintained by 120 h after 600APAP. CK19/HNF4α double-positive cells were not found. Together, the increased expression of EpCAM, A6, and CK19 could indicate HPC activation and biliary epithelial cell expansion after severe APAP overdose.

### 3.3. Hepatic Progenitor Cells Have Minimal Transdifferentiation to Hepatocyte During Liver Recovery After Severe APAP Overdose

Studies in C57BL/6J mice using severe APAP overdose showed significant activation of HPCs in surviving mice. However, it is not clear whether these HPCs transdifferentiate into hepatocytes and contribute to liver regeneration. To determine the contribution of HPCs to hepatocyte repopulation during liver regeneration after APAP-induced acute liver injury, we used a lineage-tracing approach. As described before, ROSA^mT-*mG*^ mice are treated with AAV8-TBG-Cre, which induces Cre-mediated excision of the tdTomato cassette and force permanent expression of membrane-targeted GFP (mGFP) specifically in the hepatocytes, while non-parenchymal cells remain tdTomato-positive [20]. The hepatocytes express mGFP (green fluorescence), while the non-parenchymal cells express tdTomato (red fluorescence) allowing tracing these cells.

**Figure 5:**
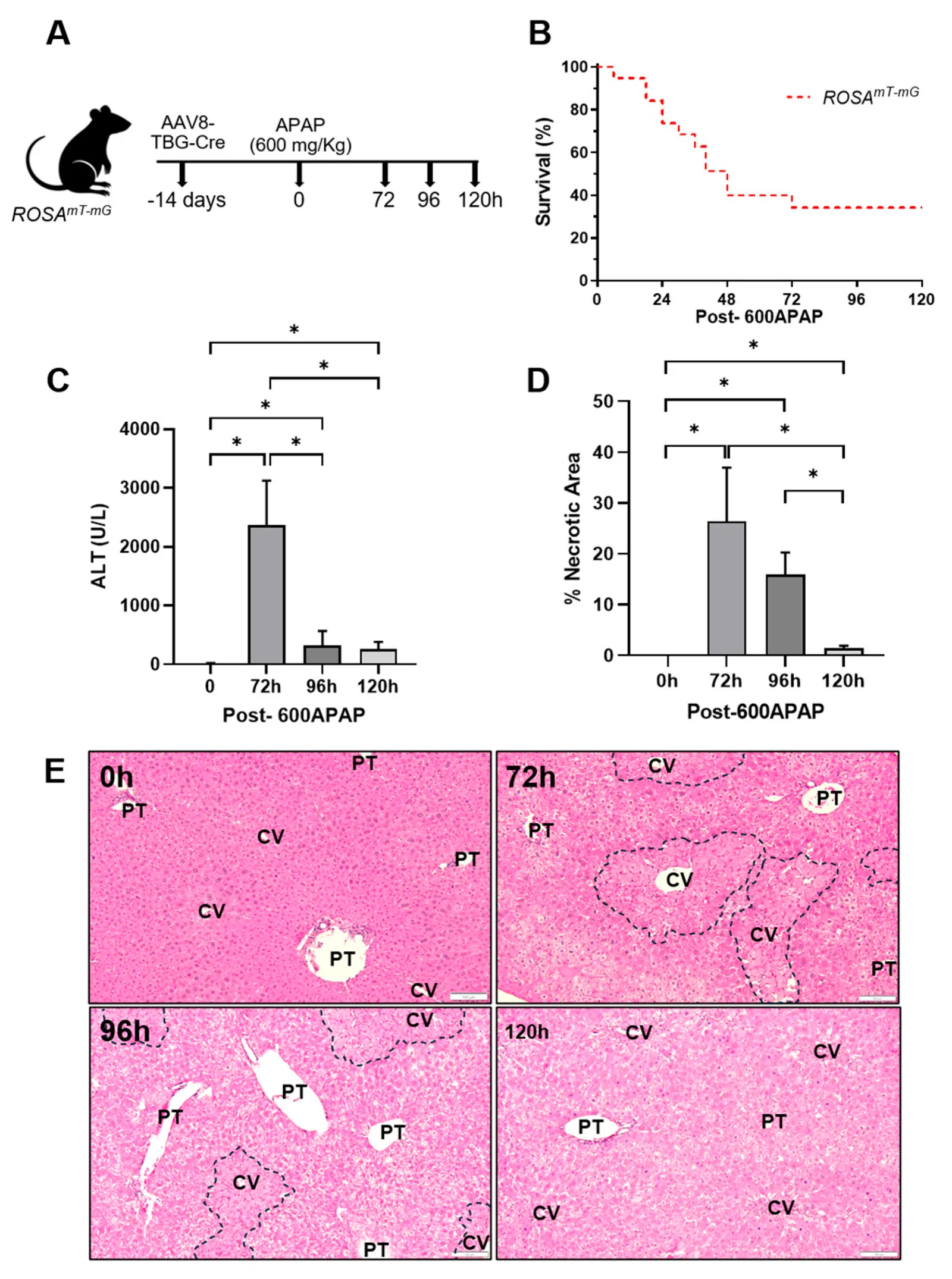
Severe liver injury after 600APAP in ROSA^mT-*mG*^ mice. (**A**) Experimental design. (**B**) Kaplan-Meier survival analysis. (**C**) Serum ALT activity. (**D**) Quantification of liver necrosis from H&E-stained liver sections. (**E**) Representative micrographs of H&E-stained liver sections. Black dashed lines indicate necrotic areas. Portal trials (PT) and central veins (CV) are indicated. Scale bar = 100 µm. Samples analyzed per group: *n* = 4 at 0 h, *n* = 4 at 72 h, *n* = 4 at 96 h, and n = 5 at 120 h. Bars represent mean ± SD, and * *p* < 0.05.

**Figure 6:**
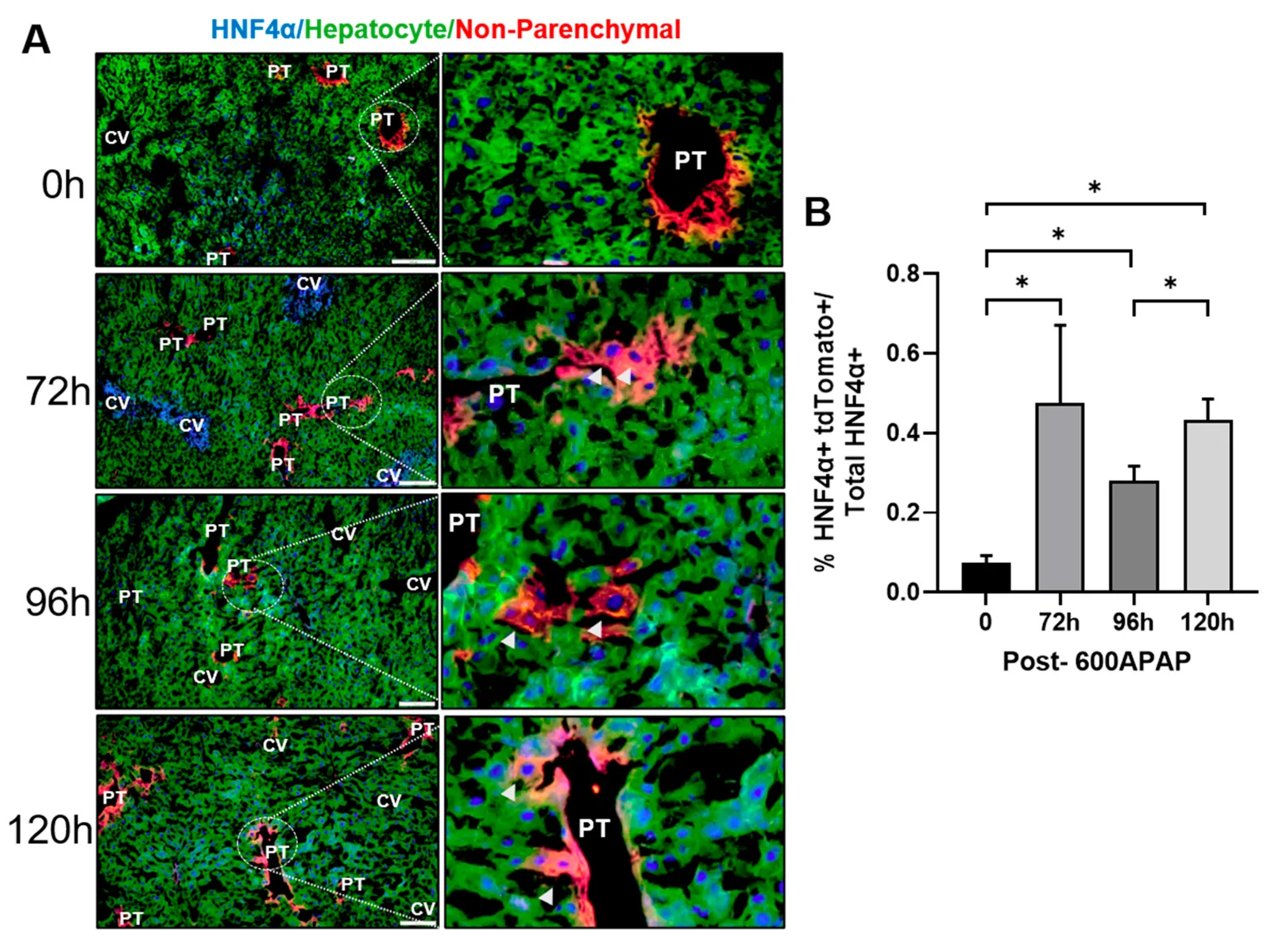
Minimal transdifferentiation of hepatic progenitor cells into hepatocytes after 600APAP. (**A**) Representative micrographs of liver sections from the lineage-tracing model (ROSA^mT-*mG*^ mice) showing hepatocytes expressing GFP (green) and non-parenchymal cells expressing tdTomato (red) including portal trials region magnification (15,000× magnification). HNF4α immunofluorescence staining is shown in blue. Portal trials (PT) and central veins (CV) are indicated. Scale bar = 100 µm. (**B**) Quantification of HNF4α/tdTomato double-positive cells and total HNF4α-positive cells in liver sections. Samples analyzed per group: *n* = 4 at 0 h, *n* = 4 at 72 h, *n* = 4 at 96 h, and *n* = 4 at 120 h. Bars represent mean ± SD, and * *p* < 0.05.

Next, the ROSA^mT-*mG*^ mice were treated with 600APAP, and liver tissue and serum were collected to evaluate injury at multiple time points (Figure 5A). We observed that 34% of the mice survive after 120 h, which is similar survival to wild type mice (Figure 5B). Serum ALT levels increased to 2363.39 ± 658.21% U/L at 72 h after 600APAP and decreased to 96 h and 120 h (Figure 5C). Histological analysis showed 26.37 ± 9.14% necrotic area at 72 h (Figure 5D,E). Necrosis decreased to 15.9 ± 3.7% at 96 h and was nearly resolved by 120 h after 600APAP, with only 1.4 ± 0.42% necrotic area remaining (Figure 5D,E). These results demonstrate that 600APAP induces comparable liver injury in ROSA^mT-*mG*^ mice and C57BL/6J mice. To determine the percentage of non-parenchymal cells (NPCs) that transdifferentiated into hepatocytes following severe APAP-induced liver injury, we performed immunofluorescence staining for the hepatocyte marker HNF4α. NPCs-derived transdifferentiated hepatocytes were identified by the co-expression of HNF4α and tdTomato. HNF4α/tdTomato double-positive cells increased to 0.48% of total HNF4α-positive hepatocytes at 72 h post-APAP, decreased to 0.28% at 96 h, and at 120 h we found at 0.43% (Figure 6A,B). Despite this increase, NPC-derived hepatocytes contributed minimally to hepatocyte replenishment, representing only ~3–6 cells per 200× field relative to the total HNF4α-positive hepatocyte population after APAP-induced liver injury.

## 4. Discussion

The liver is well known for its exceptional regenerative capacity [3,11,21]. Liver regeneration is conducted by the proliferation of remaining hepatocytes followed by non-parenchymal cells [1,21]. However, during severe liver injury, either due to progressive diseases or massive loss of hepatocytes due to chemical exposure, the ability of the liver to regenerate fails [5,15]. In these cases, hepatic progenitor cells are activated, or biliary epithelial cells undergo proliferation and then differentiation into hepatocytes [4,5,17,22]. However, most studies indicate that HPCs make only a limited contribution to hepatocyte replenishment during liver regeneration [23,24,25].

Previous studies have investigated activation of HPCs and expansion of biliary epithelial cells after APAP-induced liver injury [26], but whether the activated and proliferating HPCs contribute to regeneration by undergoing transdifferentiation has not been studied [27]. Our study corroborated previous results and showed increased expression of markers such as EpCAM, A6, and CK19 after severe APAP overdose, which indicates activation of HPCs and/or expansion of cholangiocytes after liver injury. However, lineage tracing using the ROSA^mT-*mG*^ mice revealed that HPC/cholangiocyte-derived hepatocytes represented less than 1% of the total hepatocyte population.

Our observation of periportal HPC/cholangiocyte activation after APAP overdose is consistent with Kofman et al. (2005), who described a dose- and time-dependent oval cell reaction confined to the smallest portal tracts, consistent with derivation from the canals of Hering [26]. EpCAM is a marker of immature hepatic progenitors and biliary epithelial cells, reflecting early progenitor activation [28]. A6 identifies reactive ductular cells and activated progenitors [22], whereas CK19 is a cholangiocyte marker that increases during the ductular reaction [22]. CK19+ elongated cells extending from biliary ducts into sinusoids that we observed are characteristic of the ductular reaction, a phenomenon widely associated with HPC activation across injury models [29,30]. Similar HPCs reactions have been identified after sublethal APAP doses in mice and in APAP-induced ALF patients with severe necrosis [6,26]. Additionally, the transient increase in EpCAM and sustained A6 and CK19 expression is associated with HPC proliferation rather than cell transdifferentiation to hepatocytes, suggesting roles beyond hepatocyte replacement.

Multiple studies have shown the role of hepatocytes in liver recovery during APAP-induced liver injury [11,13,15,31,32]. For example, increased PCNA, Ki67, and BrdU staining show hepatocyte proliferation surrounding the necrotic area beginning at 12–24 h post APAP [31,33,34]. scRNA-seq and spatial transcriptomics have identified proliferating hepatocytes as a distinct population that emerges in a zonal and time-dependent pattern after APAP injury [35]. Additionally, a study combining snRNA-seq, spatial profiling, and 4D intravital imaging identified ANXA2+ migratory hepatocytes as mediators of the closure of the necrotic area [35].

The minimal HPC/cholangiocyte to hepatocyte transdifferentiation we observed aligns with accumulating lineage-tracing evidence that progenitor contribution to hepatocyte regeneration is limited, particularly in acute liver injury [23,24,25]. Clonal labeling of Sox9+ ductal progenitors across multiple oval cell injury models (CDE, DDC, and CCl_4_) has demonstrated <1% contribution to the hepatocyte pool, with hepatocyte chimera models confirming that non-parenchymal-derived hepatocytes remain below ~1% and are not required for survival or liver function [23]. Complementary fate-tracing studies have demonstrated that hepatocyte self-duplication, rather than progenitor cell differentiation, is the predominant regenerative mechanism in these models [24,25].

Acute liver injury and subsequent acute liver failure following APAP overdose are hyperactive clinical phenomena which occur extremely fast, from 3 to 96 h post-overdose from initiation of injury to resolution of injury in mice [36], and from approximately 12 h to 10 days in humans [37]. The rapid progression and resolution of APAP-induced liver injury may limit the time required for HPCs to transdifferentiate to hepatocytes, which is more commonly observed during chronic liver injury. Further, the limited transdifferentiation observed after APAP overdose may also reflect the spatial organization of liver injury. APAP causes centrilobular necrosis [38], whereas HPCs reside within the periportal canals of Hering located close to bile ducts [6]. This anatomical separation and the rapid regenerative response of surviving hepatocytes limit the migration and differentiation of HPCs into injured regions. In contrast, chronic liver diseases generate sustained cell cycle arrest, inflammation, and tissue remodeling that favor progenitor expansion and migration of HPCs in these models [39,40].

Whereas HPCs/cholangiocytes do not meaningfully replace hepatocytes, their activation may serve as alternative functions. The ductular reaction is increasingly recognized as paracrine signaling process [41,42]. The CK19+ sinusoidal cells we observed may contribute to the secretion of cytokines and chemokines that modulate inflammatory cell recruitment and hepatocyte proliferation [41,42]. Thus, HPC activation or biliary epithelial cell expansion may represent a default response to severe injury, increasing with damage regardless of hepatocyte regenerative capacity [43,44]. Notably, cells expressing progenitor markers during injury may arise from dedifferentiation of mature hepatocytes or biliary epithelial cells rather than from a distinct stem cell niche [27,41].

Several limitations of our study should be acknowledged. Although EpCAM, A6, and CK19 are established HPC markers, none of them are completely specific, and they can also label mature cholangiocytes, ductular reaction cells, or reprogrammed hepatocytes [5,27,45]. This marker overlap complicates the design of lineage-tracing strategies aimed at capturing HPCs specifically. In addition, analysis after APAP overdose following 600 mg/kg dose is subject to survival bias, as only mice with favorable outcomes survived for evaluation. Therefore, the contribution of HPCs to non-surviving mice with more severe injury and impaired regeneration remains unknown. Overall, our findings demonstrate that severe APAP overdose induces HPC response and cholangiocyte expansion; however, this activation contributes minimally to hepatocyte repopulation.

## 5. Conclusions

Our findings demonstrate that severe acetaminophen overdose induces HPC activation and biliary epithelial cell expansion during recovery after severe liver injury when regeneration is impaired. However, lineage-tracing analysis revealed that HPC/cholangiocyte-derived cells account for less than 1% of total hepatocytes, indicating a minimal contribution to hepatocyte replenishment even under conditions of severe injury and impaired hepatocyte regeneration by acetaminophen.

## Figures and Tables

**Table 1:** Initial number of mice and number of survivors at each time point.

	Strain			
Time Point (h)	C57BL6/J		ROSA^mT-*mG*^	
	Initial	Surviving	Initial	Surviving
0	4	4	4	4
72	10	4	12	4
96	15	5	12	4
120	12	4	15	5

**Table 2:** Immunohistochemistry antibodies.

Antibody	Catalog Number, Source	Dilution
Human HNF4 alpha	PP-H1415-0C, Perseus Proteomics, Tokyo, Japan	1:1000
Anti-Cytokeratin 19 [EP1580Y]	ab52625, Abcam Inc., Waltham, MA, USA	1:600
Biotin-SP AffiniPure^®^ Goat Anti-Mouse IgG	115-065-072, Jackson ImmunoResearch Inc., West Grove, PA, USA	1:500
Biotin-SP AffiniPure^®^ Goat Anti-Rabbit IgG (H + L)	111-065-003, Jackson ImmunoResearch Inc., West Grove, PA, USA	1:500

**Table 3:** Immunofluorescence antibodies.

Antibody	Catalog Number, Source	Dilution
Human HNF4 alpha	PP-H1415-0C, Perseus Proteomics, Tokyo, Japan	1:1000
CK19	ab52625, Abcam Inc., Waltham, MA, USA	1:600
EpCAM	ab71916, Abcam Inc., Waltham, MA, USA	1:300
A6 BCM	Developmental Studies Hybridoma Bank, University of Iowa, Iowa City, IA, USA	1:100
Cy2 anti-rat	112-225-175, Jackson ImmunoResearch Inc., West Grove, PA, USA	1:500
Cy2 anti-rabbit	211-222-171, Jackson ImmunoResearch Inc., West Grove, PA, USA	1:500
AMCA AffiniPure^®^ Donkey Anti-Mouse IgG (H + L)	715-155-150, Jackson ImmunoResearch Inc., West Grove, PA, USA	1:50

## Data Availability

The data presented in this study are available within the article.
